# Complicated acute type A aortic dissection and severe aortic atherosclerosis predict early mortality after frozen elephant trunk procedure

**DOI:** 10.1093/ejcts/ezaf213

**Published:** 2025-06-27

**Authors:** Christian Detter, Lennart Bax, Giuseppe Panuccio, Tilo Kölbel, Yskert von Kodolitsch, Hermann Reichenspurner, Till Joscha Demal, Jens Brickwedel

**Affiliations:** Department of Cardiovascular Surgery, German Aortic Center Hamburg, University Heart & Vascular Center Hamburg, Hamburg, Germany; Department of Cardiovascular Surgery, German Aortic Center Hamburg, University Heart & Vascular Center Hamburg, Hamburg, Germany; Department of Vascular Medicine, German Aortic Center Hamburg, University Heart & Vascular Center Hamburg, Hamburg, Germany; Department of Vascular Medicine, German Aortic Center Hamburg, University Heart & Vascular Center Hamburg, Hamburg, Germany; Clinic for Thoracic and Cardiovascular Surgery, Section of Vascular Surgery, Heart and Diabetes Centre North Rhine Westphalia, Bad Gehlhausen, Germany; Department of Cardiovascular Surgery, German Aortic Center Hamburg, University Heart & Vascular Center Hamburg, Hamburg, Germany; Department of Cardiovascular Surgery, German Aortic Center Hamburg, University Heart & Vascular Center Hamburg, Hamburg, Germany; Department of Cardiovascular Surgery, German Aortic Center Hamburg, University Heart & Vascular Center Hamburg, Hamburg, Germany

**Keywords:** Aortic aneurysm, Type A aortic dissection, Aortic arch, Frozen elephant trunk

## Abstract

**OBJECTIVES:**

To analyse risk factors for early mortality and long-term survival including secondary distal aortic interventions in patients undergoing frozen elephant trunk surgery.

**METHODS:**

A retrospective single-centre study was conducted, including all 222 patients who underwent frozen elephant trunk surgery between 2010 and 2022. We used multivariable regression analysis to detect risk factors for early mortality and Kaplan–Meier analysis for long-term survival and secondary interventions. We introduce the term ‘complicated acute type A dissection’ for those patients in whom the dissection was complicated by malperfusion syndrome, aortic rupture, pre-hospital intubation or resuscitation.

**RESULTS:**

Thirty-day mortality decreased significantly from 18.9% using the conventional zone 3 technique to 7.4% using a simplified zone 2 technique (*P* = 0.014). The aortic pathology had a significant impact on 30-day mortality: 1.4% in chronic dissection, 6.7% in aortic aneurysm, 7.4% in noncomplicated acute type A aortic dissection and 42.5% in complicated acute type A aortic dissection (*P* < 0.001). We identified complicated acute type A aortic dissection [odds ratio 15.7, confidence interval (CI) 5.2–47.3, *P* < 0.001], severe aortic atherosclerosis (odds ratio 4.9, CI 1.6–15.3, *P* = 0.006) and impaired renal function (odds ratio 3.7, CI 1.1–12.4, *P* = 0.035) as independent predictors of early mortality. Among 30-day survivors, 5-year survival was 84.3%, with no differences between pathologies. Secondary distal aortic interventions (37.4%) did not affect 5-year survival (*P* = 0.909).

**CONCLUSIONS:**

Early mortality after frozen elephant trunk surgery is strongly driven by preoperative patient condition, particularly in the presence of complicated acute type A dissection. Once the early postoperative phase is overcome, long-term outcome is favourable across pathologies, regardless of secondary interventions. Careful patient selection and regular follow-up are crucial for optimizing outcomes.

## GRAPHICAL ABSTRACT

**Figure:**
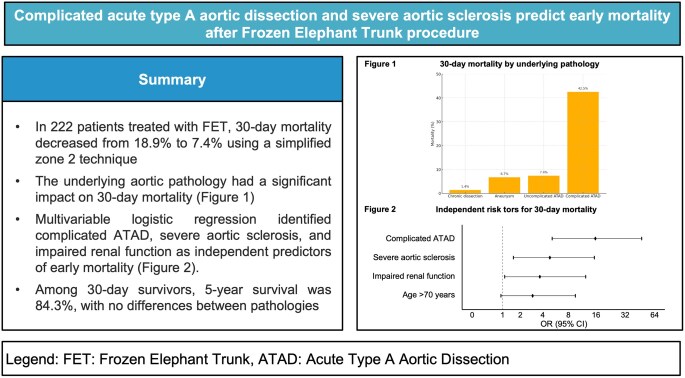

## INTRODUCTION

The frozen elephant trunk (FET) procedure has become an established single-stage procedure for the treatment of complex thoracic aortic pathologies involving the arch and the proximal descending aorta [1]. The hybrid therapy, combining conventional open surgical and endovascular techniques, has emerged as an ideal concept for patients with extensive thoracic aortic disease. In acute DeBakey type I dissection, the FET technique has been shown to promote false lumen thrombosis around the stent graft and thereby enable aortic remodelling and potentially reducing the need for secondary interventions. However, despite refinement and further development of the FET technique, early outcomes remain unsatisfactory with mortality rates up to 21% or even higher. So far, it is not well understood which clinical factors improve or deteriorate survival rates. Therefore, the aim of this study is to determine perioperative risk factors influencing early mortality and long-term survival in a single-centre cohort of patients undergoing FET for extensive aortic aneurysm or acute and chronic aortic dissection.

## METHODS

### Study design and ethics approval

The study was a single-centre, observational trial. All patients who received FET surgery between October 2010 and November 2022 were extracted from the prospectively collected departmental aortic database. The Ethics Committee of the University Heart Center Hamburg waived the need for ethics approval or informed consent for the use of anonymized and retrospectively analysed data. An approval for the data collection was obtained. The primary outcome was 30-day mortality. Secondary outcomes were postoperative morbidity (e.g. stroke rate), rate of secondary distal aortic interventions (endovascular or open surgical) and follow-up survival.

### Patients and surgical technique

This retrospective study includes patients who underwent total aortic arch surgery using the FET technique for acute and chronic aortic pathologies. The indication for FET was made in accordance with the applicable clinical guidelines and expert consensus recommendations valid at the time of surgery.

Our current surgical technique has been described in detail previously [2].

The neuroprotection protocol is based on moderate hypothermic circulatory arrest and bilateral selective antegrade cerebral perfusion with cerebral perfusion monitoring by near-infrared spectroscopy. All patients are cooled to a target core temperature of 25°C.

In 2015, our group implemented a simplified FET technique in arch zone 2. This technique includes arterial cannulation of the left subclavian artery (LSA) via an 8-mm Dacron graft using a left-sided supraclavicular approach. This approach enables perfusion of the supra-aortic vessels including the LSA and both vertebral arteries. Thus, near-physiological perfusion of both the brain and the upper spinal cord is maintained. After the distal graft anastomosis of the Thoraflex™ prosthesis was performed in zone 2, cardiopulmonary bypass (CPB) was restarted using the perfusion side branch of the prosthesis to provide early antegrade lower body perfusion. This technique allows for significantly shortened circulatory arrest times, further enhancing spinal cord protection and improving overall procedural safety.

### Definitions

Impaired renal function was defined as a creatinine clearance <50 ml/min.

Severe aortic atherosclerosis was defined as pronounced calcium deposits with extensive intimal thickening of ≥4mm, ulcerated or atherothrombotic lesions (shaggy aorta) in the ascending aorta and aortic arch including the origin of the supra-aortic vessels (typically the brachiocephalic trunk) based on the individual computed tomography (CT) scans and the corresponding surgical reports [3].

A complicated acute type A dissection (ATAD) was defined by the presence of one or more of the following high-risk features:

- Malperfusion syndrome, defined as the presence of imaging evidence of malperfusion and clinical evidence of end-organ ischaemia related to inadequate aortic branch vessel perfusion resulting in end-organ injury characterized by necrosis and/or organ dysfunction [1, 4]. The TEM classification was used to describe the location of the malperfused ischaemic organ [1].
- Free or contained aortic rupture
- Intubation prior to the anesthesiologic induction of surgery, or
- Preoperative cardiopulmonary resuscitation.

### Follow-up

All patients underwent CT or magnetic resonance imaging (MRI) with 3D reconstruction at discharge or 3 months after surgery and annually thereafter, including clinical evaluation at our specialized outpatient aortic centre. Further information on patient status and the occurrence of postoperative complications was obtained through digital medical records.

### Statistical analysis

Baseline characteristics and procedural outcomes were summarized descriptively using frequencies and percentages for categorical variables or median and interquartile range (IQR) for continuous variables. Binary outcome parameters were compared between subgroups using the Chi-squared test or Fisher’s exact test, where applicable and continuous variables were compared using the Mann–Whitney U-test. Risk factors for 30-day mortality were identified using univariable and subsequent multivariable binary logistic regression models with stepwise backward elimination (Wald) to account for the small sample size and the limited number of events. Tested parameters were age >70 years, preoperatively impaired renal function with a creatinine clearance below 50 ml/min, noncomplicated ATAD, severe aortic atherosclerosis, concomitant coronary artery bypass grafting (CABG), hereditable thoracic aortic disease (HTAD) and use of the simplified FET technique. Variables with *P* > 0.25 in univariable analysis were not included in the multivariable analysis. Adjusted odds ratios (ORs), 95% confidence intervals (CIs) and *P*-values were reported from these models. To assess the calibration of the final logistic regression model, the Hosmer–Lemeshow goodness-of-fit test was applied, comparing observed and expected event rates across quintiles of predicted risk. A non-significant result (*P* > 0.05) was interpreted as indicative of adequate model fit. Discrimination was assessed by calculating the area under the receiver operating characteristic curve (AUC) using the model’s predicted probabilities. The AUC and its 95% CI quantified the model’s ability to distinguish between patients who did and did not experience 30-day mortality (Supplementary Material, Fig. S1). For estimation of long-term survival, Kaplan–Meier analysis and log-rank tests were performed. A competing risk analysis was performed for freedom from secondary distal aortic intervention, and group differences were assessed using Gray’s test. The level of significance for all analyses was set at α = 0.05. Follow-up time was estimated using the reverse Kaplan–Meier method. The follow-up rate (completeness of the latest annual outpatient follow-up before end of data procurement, i.e. 20.12.2023) was calculated using the Simplified Person-Time method as proposed by Xue *et al.* [5].

Statistical analyses were performed using IBM SPSS version 29.0.1.0 (IBM, Armonk, New York, USA), R version 4.5.0 (for competing risk analysis only) and GraphPad Prism version 10.2.3.

## RESULTS

### Patient characteristics

Between October 2010 and November 2022, 222 consecutive patients underwent total aortic arch surgery using the FET technique. The Thoraflex hybrid-graft (Terumo Aortic, Inchinnan, UK) was implanted in 207 patients (93.2%) and the E-vita open (Jotec, Hechingen, Germany) in the first 14 FET patients (6.8%). A conventional FET procedure was performed in 74 patients and the simplified FET technique in 148 patients (66.7%) (Supplementary Material, Tables S5–S7). Median age was 63.8 (IQR: 53.9–72.2) years, and 135 (60.8%) patients were male. The underlying pathologies were aortic aneurysm (33.8%), chronic type A or B aortic dissection (31.5%), ATAD (30.2%) or acute type B aortic dissection (4.5%). In 40 patients (18.0%), ATAD was complicated by organ malperfusion (*n* = 31), aortic rupture (*n* = 15), intubation before arrival at our centre (*n* = 6) and/or preoperative resuscitation (*n* = 2) (Supplementary Material, Table S1). Preoperative neurological deficit on admission (*n* = 22; 9.9%) and spinal cord ischaemia (*n* = 8; 3.6%) were found exclusively in patients with ATAD. Of the 47 patients with HTAD, 24 had genetically confirmed Marfan syndrome, 6 Loeys–Dietz syndrome, 1 Ehlers Danlos syndrome and 16 patients had other genetic mutations (e.g. ACTA II). HTAD patients were significantly younger than other patients [46.9 (IQR 37.5–54.7) vs 66.9 (IQR 59.7–73.5) years; *P* < 0.001, r = 0.6]. In total, 46 patients (20.7%) had previous aortic (*n* = 44) or CABG (*n* = 2) surgery.

Median follow-up was 1.9 years (IQR: 1.6–2.2), and follow-up rate was 67.7%.

Patient and procedural characteristics are presented in Tables 1 and 2 (for more detailed information about patient and procedural characteristics, as well as postoperative outcomes in HTAD patients, see Supplementary Material, Tables S2–S4).

**Table 1:** Patient characteristics

Variable	*n* = 222 (%)
Male	135 (60.8)
Age (years), median (IQR)	63.8 (53.9–72.2)
Age >70 years	64 (28.8)
Arterial hypertension	207 (93.2)
HTAD	47 (21.2)
Diabetes	13 (5.9)
Smoking	80 (36.0)
COPD	44 (19.8)
Peripheral vascular disease	90 (40.5)
Severe aortic atherosclerosis	66 (29.7)
Impaired renal function^a^	25 (11.3)
Preoperative impaired neurological status	22 (9.9)
Preoperative spinal cord ischaemia	8 (3.6)
Prior open cardiac or aortic surgery	46 (20.7)
Pathologies	
Thoracic aortic aneurysm	75 (33.8)
Noncomplicated acute type A dissection	27 (12.2)
Complicated acute type A dissection	40 (18.0)
Acute type B dissection	10 (4.5)
Chronic dissection (A: 44/B: 26)	70 (31.5)

a Creatinine clearance <50 ml/min. COPD: chronic obstructive pulmonary disease; HTAD: hereditable thoracic aortic disease; IQR: interquartile range.

**Table 2:** Procedural characteristics

Variable	*n* = 222 (%)
E-vita open hybrid	15 (6.8)
Thoraflex hybrid	207 (93.2)
Simplified FET	148 (66.7)
Ascending procedures	
Supracoronary aortic replacement	178 (80.2)
Bentall procedure	25 (11.3)
Valve-sparing root replacement	18 (8.2)
Aortic valve replacement	36 (16.3)
Coronary artery bypass grafting	23 (10.4)
Tricuspid valve repair	8 (3.6)
Mitral valve repair	3 (1.4)
Cardiopulmonary bypass time (min), median (IQR)	243 (203–303)
Cross-clamp time (min), median (IQR)	112 (93–152)
Circulatory arrest time (min), median (IQR)	44 (36–60)
Cerebral perfusion time (min), median (IQR)	67 (57–80)
Lowest body temperature (°C), median (IQR)	25 (24–25)

FET: frozen elephant trunk; IQR: interquartile range.

### Early outcomes

**Figure 1:**
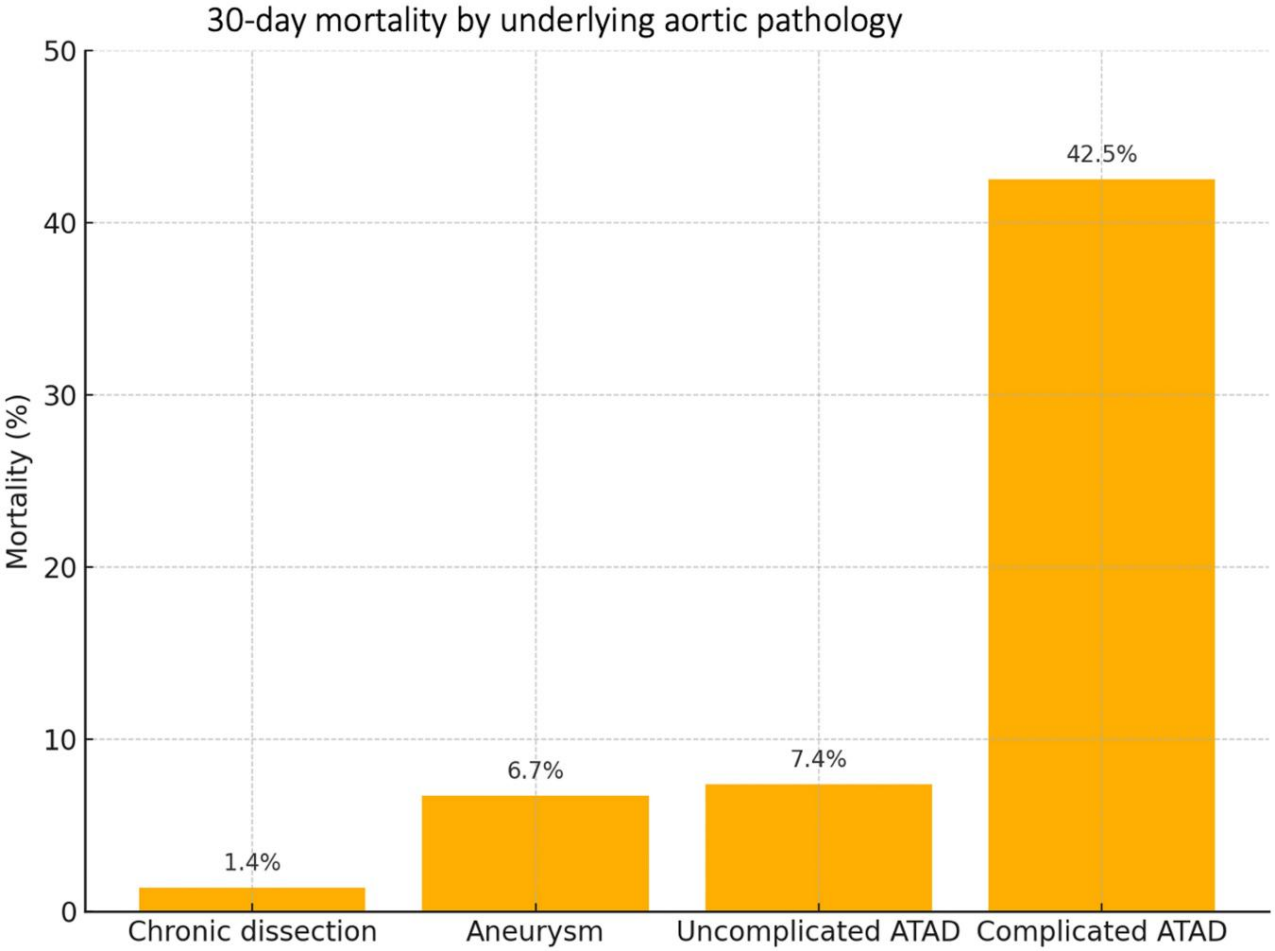
Thirty-day mortality by underlying aortic pathology. Thirty-day mortality was 1.4% in chronic dissections and 6.7% in aortic aneurysms. In ATAD, 30-day mortality differed profoundly with 7.4% in uncomplicated ATAD and 42.5% in complicated ATAD.

The overall 30-day mortality rate was 11.3% (25/222) and significantly decreased from 18.9% using the conventional FET technique in zone 3 compared to 7.4% since using the simplified technique (*P* = 0.014). Thirty-day mortality by aortic pathology is shown in Fig. 1. The overall 30-day mortality according to the aortic pathology was 1.4% in chronic dissection, 6.7% in aortic aneurysm, 7.4% in noncomplicated ATAD and 42.5% in complicated ATAD (42.5% vs 7.4%, *P* < 0.001). Among the 17 patients who died after surgery for complicated ATAD, 6 were operated on in a bailout situation due to aortic rupture, 14 had preoperative severe malperfusion, 2 received preoperative cardiopulmonary resuscitation and were intubated at admission (Supplementary Material, Table S1). The 30-day mortality rate was 2.1% (1/47) in HTAD patients and 4.3% in redo FET procedures.

New postoperative stroke was observed in 20 (9.0%) patients (Table 3). The stroke rate was significantly higher in complicated ATAD compared to elective procedures (27.5% vs 4.1%; *P* < 0.001) and in patients with severe aortic atherosclerosis compared to elective procedures without presence of aortic atherosclerosis (15.2% vs 1.0%; *P* < 0.001). The 30-day mortality rate was significantly higher in patients who suffered from stroke compared to patients without stroke (40.0% vs 8.4%, *P* < 0.001). Spinal cord injury occurred in 7 patients (3.2%) and recurrent nerve palsy in 32 patients (14.4%). Among the latter, 8 patients had previous aortic surgery and 19 patients underwent conventional FET in zone 3.

**Table 3:** Postoperative complications

Variable	*n* = 222 (%)
Transient neurological deficit	27 (12.2)
Permanent neurological deficit	20 (9.0)
Recurrent nerve palsy	32 (14.4)
Spinal cord ischaemia	3 (1.4)
Postoperative renal failure	36 (16.2)
Resternotomy for bleeding	24 (10.8)

### Predictors for early mortality

Univariable logistic regression analysis for 30-day mortality revealed age >70 years (OR 3.1, CI 1.3–7.2, *P *= 0.009), impaired renal function (OR 5.0, CI 1.9–13.2, *P *= 0.001), complicated ATAD (OR 14.0, CI 5.5–35.6, *P *< 0.001), concomitant CABG (OR 3.3, CI 1.2–9.4, *P *= 0.025) and severe aortic atherosclerosis (OR 6.4, CI 2.6–15.7, *P *< 0.001) as predictors of 30-day mortality after FET procedure. Patients with HTAD showed a trend towards lower early mortality (OR 0.1, CI 0.0–1.0, *P *= 0.054) while noncomplicated ATAD did not affect mortality (OR 0.6, CI 0.1–2.7, *P* = 0.5) and was thus not added to the multivariable model. The simplified FET technique was associated with improved early outcome (OR 0.3, CI 0.1–0.8, *P *= 0.013) (Table 4).

**Table 4:** Univariable and multivariable logistic regression analysis for 30-day mortality

	Univariable analysis			Multivariable analysis		
Variable	OR	95% CI	*P*-value	OR	95% CI	*P*-value
Age >70 years	3.101	1.330–7.234	0.009	2.975	0.925–9.573	0.07
Preoperatively impaired renal function^a^	4.983	1.877–13.229	0.001	3.690	1.099–12.396	0.04
*Noncomplicated acute type A dissection* ^b^	*0.598*	*0.133*–*2.694*	*0.50*			
Complicated acute type A dissection	16.076	6.241–41.409	<0.001	15.698	5.214–47.260	<0.001
Severe aortic atherosclerosis	6.375	2.591–15.696	<0.001	4.891	1.562–15.315	0.006
Concomitant CABG	3.307	1.164–9.392	0.03			
HTAD	0.136	0.018–1.032	0.05			
Simplified FET	0.344	0.148–0.802	0.01			

a Creatinine clearance <50 ml/min.

b *Noncomplicated acute type A dissection not included into multivariable analysis, as P > 0.25.*

CI: confidence interval; FET: frozen elephant trunk; HTAD: hereditable thoracic aortic disease; OR: odds ratio.

**Figure 2:**
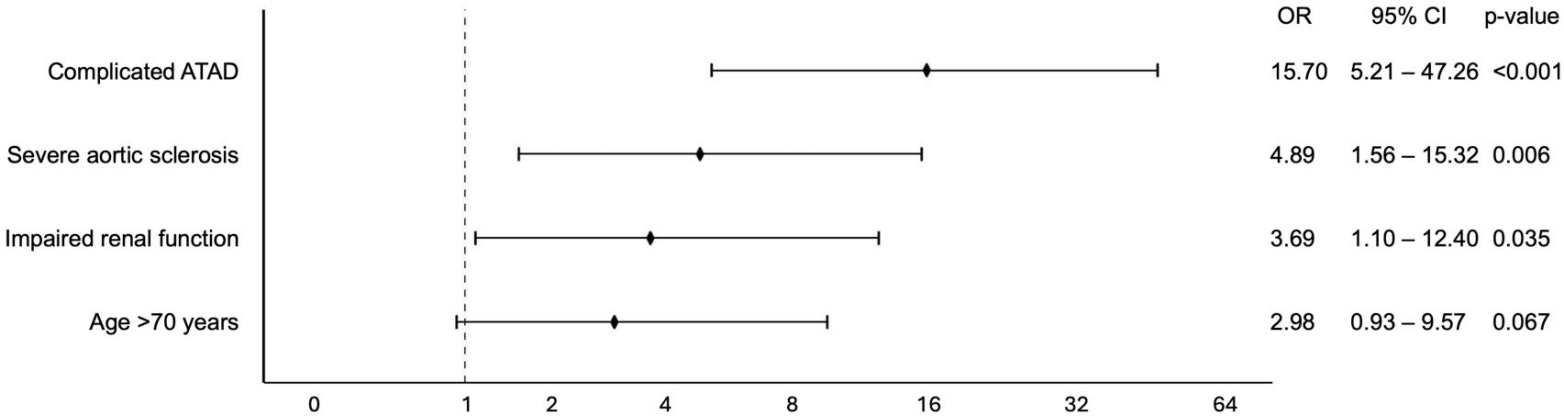
Forest plot of the multivariable logistic regression analysis for early mortality. Complicated ATAD (OR 15.70), severe aortic sclerosis (OR 4.89), and impaired renal function (OR 3.69) were identified as independent predictors for 30-day mortality.

Multivariable analysis identified complicated ATAD (OR 15.7, CI 5.2–47.3, *P* < 0.001), the presence of severe aortic atherosclerosis (OR 4.9, CI 1.6–15.3, *P* = 0.006) and preoperatively impaired renal function (OR 3.7, CI 1.1–12.4, *P* = 0.035) as independent predictors for early mortality. In addition, age >70 years showed a trend towards impaired postoperative survival (OR 3.0, CI 0.9–9.6, *P* = 0.067) (Table 4, Fig. 2). The final logistic regression model showed good calibration, as indicated by a non-significant Hosmer–Lemeshow test (χ^2^ = 4.829, df = 4, *P* = 0.305). The model also demonstrated excellent discrimination, with an AUC of 0.901 (95% CI: 0.850–0.952; *P* < 0.001).

### Long-term survival

**Figure 3:**
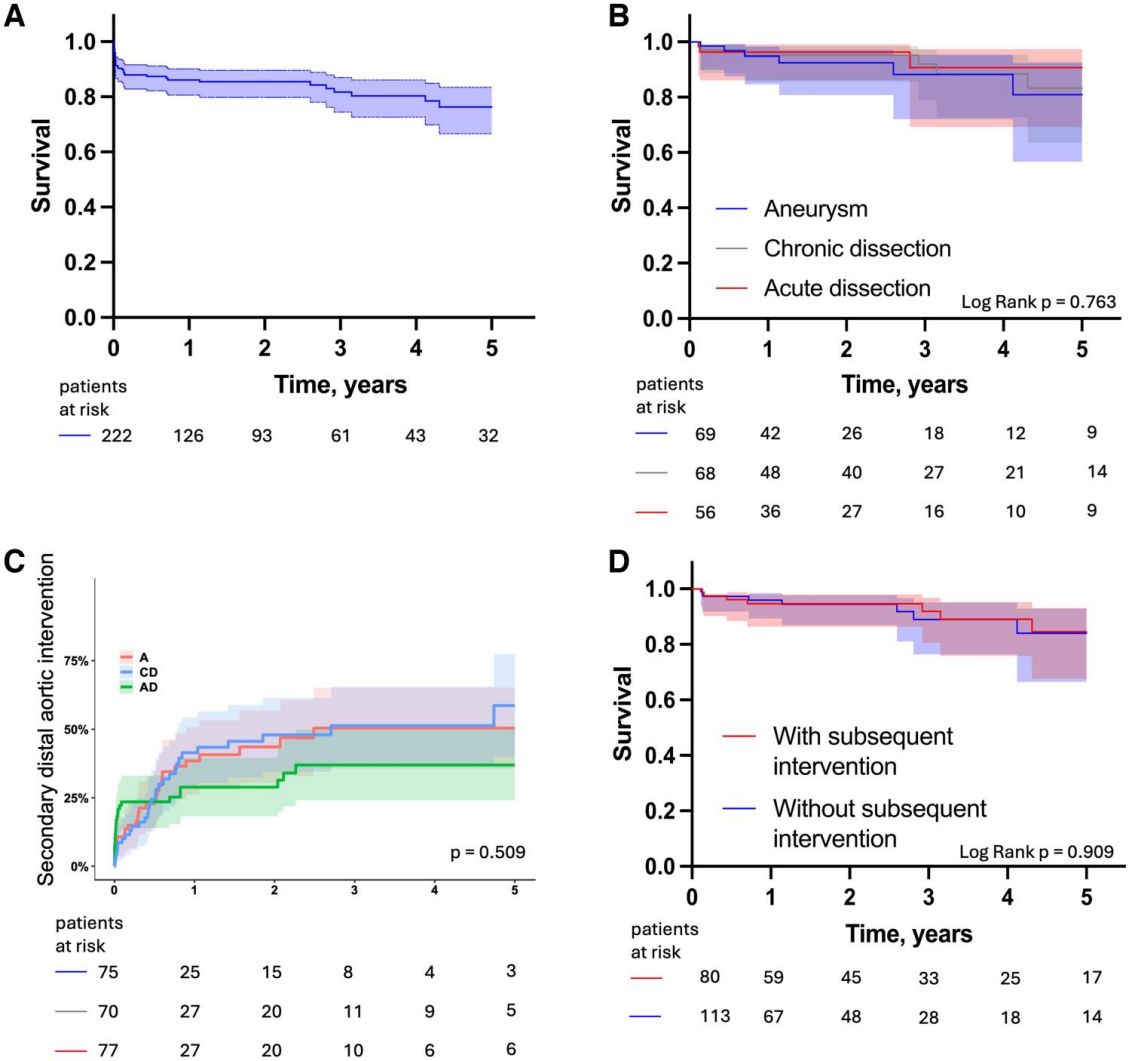
Kaplan–Meier analysis on long-term survival and secondary distal aortic interventions. (**A**) Long-term survival of the overall cohort. (**B**) Thirty-day landmark analysis on long-term survival according to the underlying aortic pathology with no statistically significant differences between the groups, Log Rank *P* = 0.763. (**C**) Rate of secondary distal aortic interventions according to the underlying aortic pathology with no statistically significant differences between the groups, competing risk analysis, *P* = 0.509. A: aneurysm; AD: acute dissection; CD: chronic dissection. (**D**) Thirty-day landmark analysis on long-term survival according to the status of secondary distal aortic intervention with no statistically significant difference between the groups, Log Rank *P* = 0.909.

In patients who survived the first 30 days, 1- and 5-year survival rates were 94.8% and 80.9% for patients with aortic aneurysm, 95.2% and 82.9% in chronic aortic dissection, and 96.3% and 90.7% in acute aortic dissection, without significant differences between the various pathologies (log rank *P* = 0.76; Fig. 3).

### Secondary distal aortic interventions

Secondary distal aortic interventions were performed in 83 (37.4%) patients within 5 years after FET as a staged approach for completion of the aortic repair or due to disease progression during follow-up and was highest in chronic dissections (Supplementary Material, Table S8). Freedom from distal aortic intervention at 5 years was 49.0% for aortic aneurysm, 41.7% for chronic aortic dissection and 64.2% for acute aortic dissection, without significant differences between groups (Gray’s test, *P* = 0.509). After surviving the early postoperative period, 5-year survival after FET surgery was not significantly different between patients with and without secondary distal aortic interventions (84.6% vs 84.0%, log rank *P* = 0.909) (Fig. 3).

## DISCUSSION

Over the last decade, the FET procedure has become an established technique for the treatment of extensive aortic pathology involving the aortic arch and descending aorta. Despite increasing experience and improved early results using the FET technique in high-volume aortic centres, early morbidity and mortality remain high due to the complexity of the surgical technique and the extent of aortic pathology [6, 7].

In the present study, the overall early mortality was 11.3%, which is well comparable with other series [8, 9]. However, early mortality varied significantly, ranging from 1.4% in chronic dissection up to 42.5% in complicated ATAD. Especially elective patients with chronic dissection and HTAD patients who were generally younger than non-HTAD patients showed excellent early outcomes, whereas elderly patients in poor acute condition had an increased perioperative mortality risk. Furthermore, the study identified complicated ATAD, severe aortic atherosclerosis and preoperative impaired renal function as independent risk factors for early mortality.

The diagnosis of ATAD is a known predictor of poor early survival, and the role of the FET technique in increasing operative risk is still under debate. The optimal surgical approach for ATAD in emergencies remains uncertain due to persistently high early mortality and neurological injury risk [8]. In a review article by Di Marco *et al.*, as well as in the GERAADA and IRAD registries, comparable mortality rates were reported between conventional treatment and total arch replacement with or without FET in the setting of ATAD [10–12]. However, only 12% of patients in the IRAD registry and 16% of patients in GERAADA underwent total arch replacement. Using real-world all-comer data, the French National Thoraflex Registry reported an in-hospital mortality of 21% in patients with ATAD who underwent emergency FET [6]. However, Shrestha *et al.* showed that after an initial learning curve, the in-hospital mortality rate decreased from 18% to 6% in their cohort, which was mainly attributed to advanced surgical experience [13]. Most aortic centres using the FET technique in ATAD have shown similar results [7, 8, 11, 12]. Thus, the FET procedure itself does not appear to increase early mortality in ATAD when performed in experienced high-volume aortic centres.

The preoperative condition of the patient is a critical determinant influencing early postoperative outcomes. Patients presenting in a poor condition requiring emergency aortic intervention have a multiple increased risk of early mortality following FET surgery [14, 15]. Patients with ATAD complicated by malperfusion have shown significantly higher mortality rates, increasing with the number of organ systems involved [16]. The GERAADA registry and other studies have shown that mesenteric malperfusion is associated with a 3- to 4-fold increase in mortality ranging from 60 to 75% [16]. The data from the IRAD registry indicate a variability in survival rates depending on the presence and type of malperfusion syndrome, with mesenteric malperfusion correlating with the highest odds of in-hospital death, followed by coma [17]. A multicentre study by Vendramin *et al.* revealed that patients with cerebral malperfusion complicated by preoperative coma exhibited an in-hospital mortality rate of 56% [18]. The present study demonstrated a significant increase in early mortality in patients presenting with complicated ATAD (defined as malperfusion syndrome, aortic rupture, pre-hospital intubation or resuscitation) compared to noncomplicated ATAD (42.5% vs 7.4%; *P* < 0.001) and was also identified as a strong independent predictor of poor early survival (OR 15.7, 95% CI 5.2–47.3; *P* < 0.001). Dumfarth *et al.* identified coronary artery disease and complicated ATAD (defined as a composite variable including preoperative cardiopulmonary resuscitation, preoperative neurological injury or preoperative malperfusion syndrome) as independent risk factors for 30-day mortality in elderly patients undergoing surgical treatment [19].

It is evident that complicated ATAD with malperfusion syndrome is a serious condition and is associated with poor surgical outcomes. Timely resolution of malperfusion to restore organ perfusion is critical, as delays may result in irreversible organ damage or death. In patients with coronary and cerebral malperfusion, immediate surgical therapy remains the treatment of choice to prevent extensive myocardial infarction or major brain injury. ATAD complicated by mesenteric malperfusion is a devastating complication associated with an almost 5-fold increased risk of perioperative death [1, 16]. Due to the poor outcomes observed with conventional aortic repair techniques, alternative strategies such as visceral revascularization prior to open repair have been proposed [17]. Yang *et al.* demonstrated favourable results in clinically stable ATAD patients presenting with malperfusion using a staged approach. This involved upfront endovascular reperfusion by fenestration or stenting, followed by open aortic surgery at resolution of organ failure [20]. While emergency surgery remains the standard treatment in ATAD, high-risk patients—such as those with malperfusion syndrome or aortic rupture—may benefit from endovascular treatment prior to surgery to decrease early mortality. Alternatively, on-site imaging with the possibility of open surgical and endovascular therapy in a hybrid room can enhance selection of an optimal treatment algorithm [16]. Therefore, the management of these patients should be individualized based on the patient’s condition, the duration of end-organ ischaemia and the urgency of aortic intervention.

Additionally, severe aortic atherosclerosis and preoperatively impaired renal function were identified as strong independent predictors for early mortality. This observation was also reported by Martens and colleagues [14]. Tokuda *et al.* found that atherothrombotic lesions were important predictors of the occurrence of neurologic deficit after total aortic arch replacement [3]. The predominantly elderly cohort of patients with giant thoraco-abdominal aneurysms and a severe cardiovascular risk profile are at particular risk of embolic-related stroke and intestinal obstruction, which can lead to severe cerebral damage, intestinal ischaemia, and death. Furthermore, the incidence of renal failure following aortic arch surgery remains as high as 21% [21]. It is well documented that renal failure in aortic surgery is a devastating complication with a significant impact on length of hospital stay and mortality [22]. In aortic arch surgery, renal perfusion is disrupted during circulatory arrest, which can result in end-organ ischaemia and reperfusion injury. Moreover, the use of moderate or deep hypothermia prolongs the duration of cardiopulmonary bypass required for cooling and rewarming the patient. Therefore, in patients with severe aortic atherosclerosis and known chronic kidney disease, less complex procedures and interventional approaches should be discussed in a multidisciplinary aortic team, including vascular surgery, cardiology and anesthesiology.

It is essential to improve the safety of these high-risk patients by reducing the surgical invasiveness and striving for shorter operative times. Our group has previously demonstrated that simplifying the FET technique results in reduced hypothermic circulatory arrest (HCA) and selective antegrade cerebral perfusion (SACP) times. This, along with and increased surgical experience, may have contributed to the improved outcomes observed in this study and previously demonstrated [2]. Other centres have also implemented various strategies to simplify the procedure. These include proximalization of the distal anastomosis, different debranching techniques, stent bridging endovascular techniques to facilitate the supra-aortic vessel anastomoses and improved organ protection using early lower body perfusion or ‘beating heart’ aortic arch surgery [23–25].

The multivariable risk analysis for early mortality highlights the importance of careful preoperative patient selection and appropriate therapy allocation. Our study results indicate that patients with complicated ATAD, severe aortic atherosclerosis and severe comorbidities, such as impaired renal function and advanced age, are at high risk of early mortality, whereas younger patients with HTAD can expect excellent 30-day survival rates. The Thoraflex French National Registry multicentre study found that patients with a log EuroSCORE ≥20 had a 6-fold risk of in-hospital death [6]. For this high-risk group, FET procedures should not necessarily always be the first-line treatment independent from entry location. Instead, a less complex and faster primary procedure—such as hemiarch replacement with an open distal anastomosis during a short HCA—may be more appropriate. This can be followed by either staged endovascular repair or a planned secondary FET procedure [26]. Subsequent treatments, including the use of custom-made fenestrated endografts, should be performed by a specialized aortic team at a high-volume centre to ensure optimal outcomes [26, 27].

After the early postoperative period, the 5-year survival rate following FET surgery was favourable (84.3%), with no significant difference observed between the various pathologies. Secondary distal aortic interventions were frequently performed after FET (37.4%); however, this did not negatively affect 5-year survival.

### Limitations

The main limitations of our study were its retrospective nature and the limited number of patients in a single-centre cohort. For further evaluation, larger numbers of patients using European multicentre cohorts or data from national registries are needed to confirm the findings of this study. In addition, the experience gained in aortic surgery with increasing case volume may have attributed to selection bias.

## CONCLUSION

The FET technique remains a challenging surgical procedure; however, early mortality is predominantly influenced by the underlying pathology and the patient’s condition. Patients with complicated ATAD, severe aortic atherosclerosis and preoperative impaired renal function have a high 30-day mortality risk when treated with FET. The simplified FET technique was associated with improved early survival. After surviving the early postoperative period, 5-year survival is encouraging regardless of the underlying pathology. Secondary distal aortic interventions, although frequently performed, did not affect long-term survival. Appropriate patient selection, simplified surgical procedures and regular follow-up are key to improve early and late outcomes.

## Supplementary Material

ezaf213_Supplementary_Data

## Data Availability

The data underlying this article will be shared on reasonable request to the corresponding author.

## ABBREVIATIONS

ATAD: Acute type A dissection
AUC: Area under the receiver operating characteristic curve
CPB: Cardiopulmonaty baypass
CABG: Coronary artery bypass grafting
CI: Confidence interval
CT: Computed tomography
FET: Frozen elephant trunk
HCA: hypothermic circulatory arrest
HTAD: Hereditable thoracic aortic disease
IQR: Interquartile range
LSA: magnetic resonance imaging
MRI: left subclavian artery
OR: Odds ratio
SACP: antegrade selective cerebral perfusion
