# Supplementary material for: Complicated acute type A aortic dissection and severe aortic atherosclerosis predict early mortality after frozen elephant trunk procedure
Source: Eur J Cardiothorac Surg. 2025 Jun 27;67(7):ezaf213. doi: 10.1093/ejcts/ezaf213 (PMC12270253; doi:10.1093/ejcts/ezaf213)
Supplement: ezaf213_Supplementary_Data [file ezaf213_supplementary_data.zip › Supplementary Figure S1.pdf]

A)

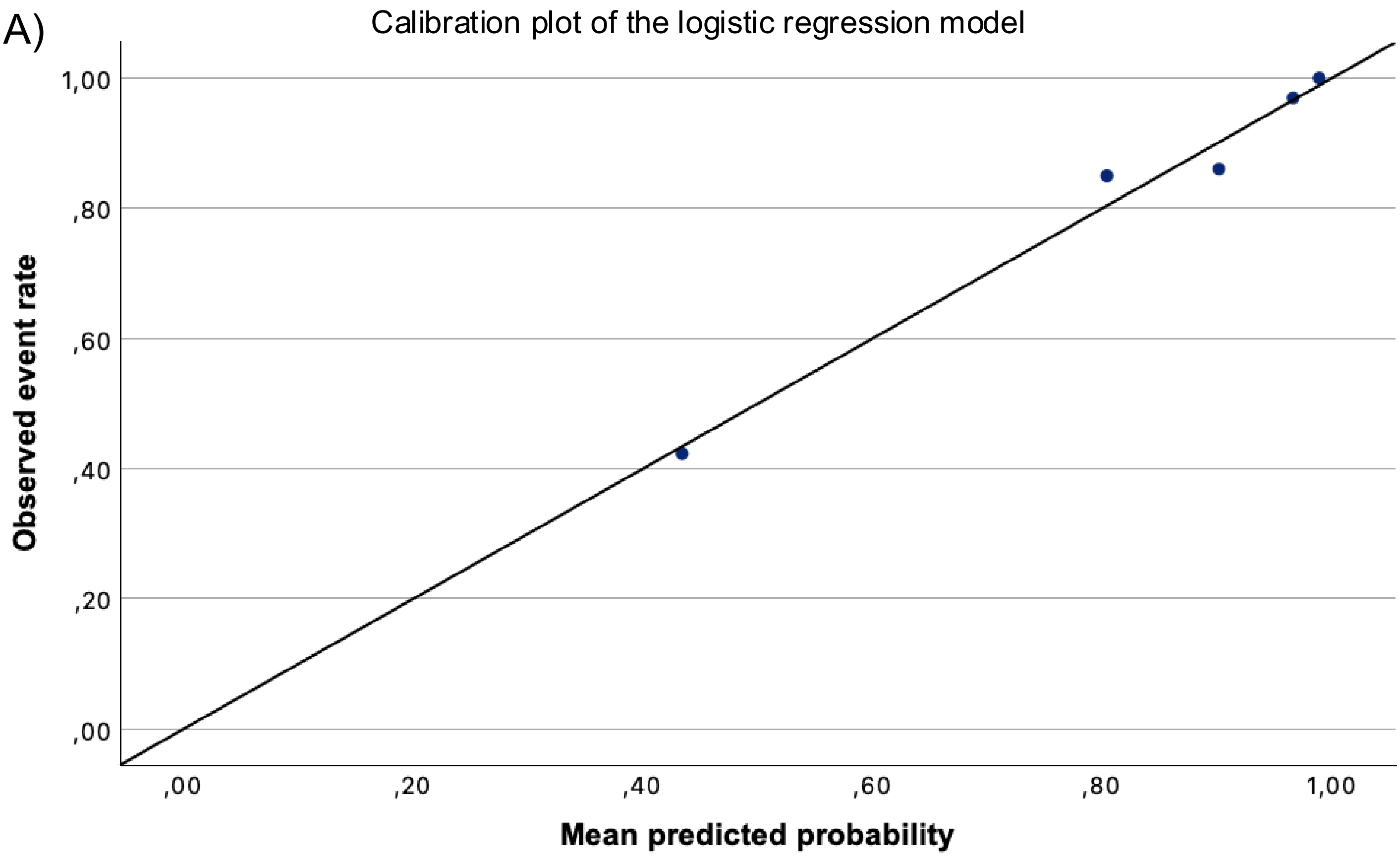

B)

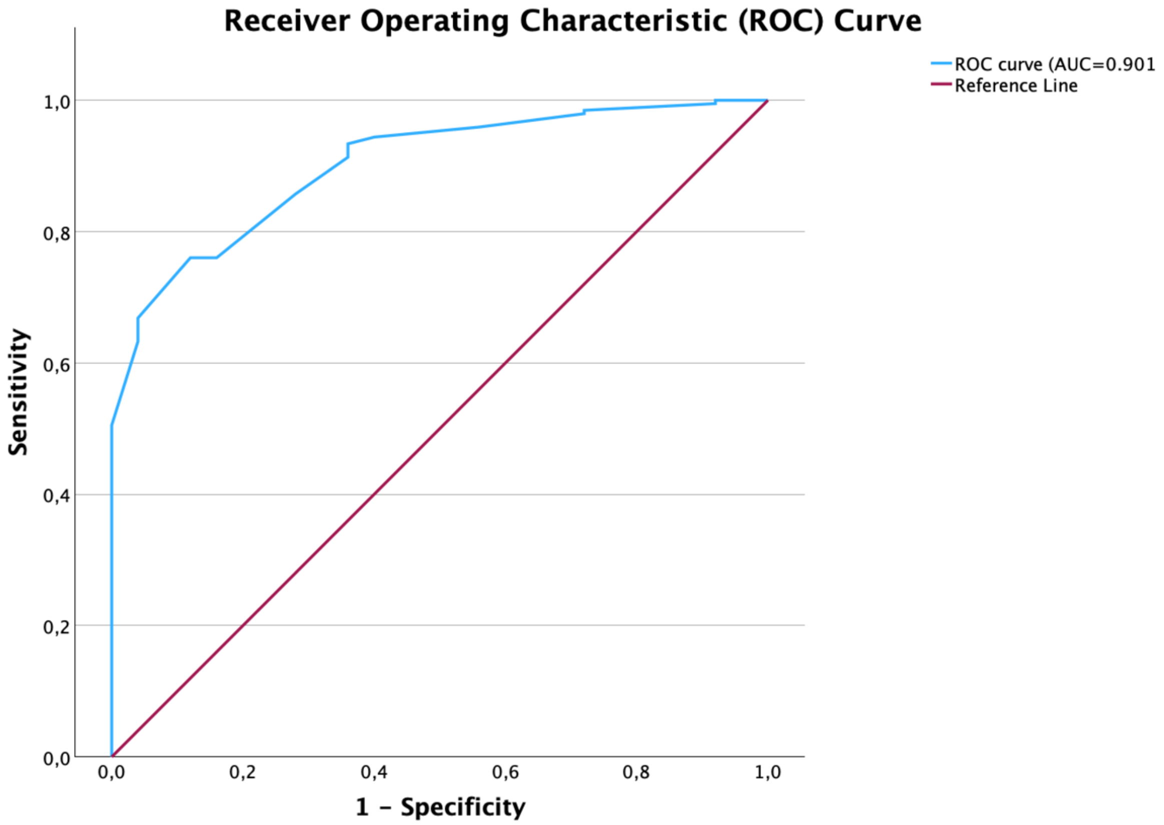

Supplementary Figure S1: Performance analyses of the logistic regression model. A) Calibration plot of the logistic regression model. The plot compares observed event rates with mean predicted probabilities across quintiles of predicted 30-day mortality risk. Each point represents one risk group. The 45-degree reference line indicates perfect agreement between predicted and observed outcomes. The model shows good calibration across the range of predicted probabilities. B) Receiver Operating Characteristic (ROC) Curve for the logistic regression model. The curve illustrates the model's ability to discriminate between patients who died and those who survived within 30 days. The area under the curve (AUC) was 0.901 (95% CI: 0.850–0.952), indicating excellent discriminative performance. The diagonal line represents the reference line for a non-informative model (AUC = 0.5).
