# Supplementary material for: Complicated acute type A aortic dissection and severe aortic atherosclerosis predict early mortality after frozen elephant trunk procedure
Source: Eur J Cardiothorac Surg. 2025 Jun 27;67(7):ezaf213. doi: 10.1093/ejcts/ezaf213 (PMC12270253; doi:10.1093/ejcts/ezaf213)
Supplement: ezaf213_Supplementary_Data [file ezaf213_supplementary_data.zip › Supplementary Table S1 to S8.docx]

Supplementary Table S1: Overview over all complicated ATAD cases

| Case No. | Malperfusion syndrome | | | | Aortic rupture | | | Preoperative intubation | Preoperative CPR | 30-day mortality |
| --- | --- | --- | --- | --- | --- | --- | --- | --- | --- | --- |
|  | Overall | M1: coronary | M2: supra-aortic | M3: spinal/ visceral/ renal/ iliacal | Overall | Free | Contained |  |  |  |
| 1 | yes | yes | no | yes | yes | yes | no | yes | yes | yes |
| 2 | no | no | no | no | yes | yes | no | no | no | no |
| 3 | no | no | no | no | yes | no | yes | no | no | yes |
| 4 | yes | yes | no | yes | no | no | no | no | no | yes |
| 5 | yes | no | yes | yes | no | no | no | no | no | yes |
| 6 | yes | yes | yes | no | no | no | no | yes | no | no |
| 7 | yes | no | yes | no | no | no | no | yes | no | no |
| 8 | yes | no | yes | no | yes | yes | no | yes | no | no |
| 9 | yes | no | yes | no | no | no | no | no | no | yes |
| 10 | yes | no | yes | no | no | no | no | no | no | no |
| 11 | yes | yes | no | no | no | no | no | no | no | yes |
| 12 | yes | yes | no | no | yes | yes | no | no | no | yes |
| 13 | yes | yes | yes | no | no | no | no | no | no | yes |
| 14 | yes | no | no | yes | no | no | no | no | no | no |
| 15 | yes | no | no | yes | yes | no | yes | no | no | no |
| 16 | no | no | no | no | yes | no | yes | no | no | no |
| 17 | no | no | no | no | yes | no | yes | no | no | yes |
| 18 | yes | yes | no | no | no | no | no | no | no | no |
| 19 | yes | no | no | yes | no | no | no | no | no | no |
| 20 | no | no | no | no | yes | no | yes | no | no | no |
| 21 | yes | yes | yes | no | no | no | no | no | no | yes |
| 22 | yes | no | no | yes | yes | yes | no | no | no | yes |
| 23 | yes | yes | yes | no | no | no | no | no | no | no |
| 24 | no | no | no | no | yes | no | yes | no | no | no |
| 25 | no | no | no | no | yes | yes | no | no | no | no |
| 26 | yes | yes | no | no | no | no | no | no | no | no |
| 27 | yes | no | no | yes | no | no | no | yes | yes | yes |
| 28 | yes | yes | yes | no | no | no | no | yes | no | no |
| 29 | yes | no | no | yes | no | no | no | no | no | yes |
| 30 | yes | no | no | yes | no | no | no | no | no | no |
| 31 | yes | no | yes | yes | no | no | no | no | no | yes |
| 32 | yes | yes | no | yes | no | no | no | no | no | no |
| 33 | yes | no | yes | yes | no | no | no | no | no | no |
| 34 | no | no | no | no | yes | no | yes | no | no | no |
| 35 | yes | no | yes | no | yes | no | yes | no | no | no |
| 36 | yes | no | yes | yes | no | no | no | no | no | no |
| 37 | yes | no | yes | yes | no | no | no | no | no | yes |
| 38 | yes | no | yes | yes | no | no | no | no | no | yes |
| 39 | no | no | no | no | yes | no | yes | no | no | yes |
| 40 | yes | yes | no | yes | no | no | no | no | no | no |
| Total | 31 | 13 | 16 | 17 | 15 | 6 | 9 | 6 | 2 | 17 |

ATAD, Acute Type A aortic Dissection; M1/2/3, Location of malperfusion as defined by TEM classification; CPR, cardiopulmonary resuscitation.

Supplementary Table S2: Patient characteristics according to presence/absence of HTAD

| Variable | Entire cohort  n = 222 (%) | HTAD  n = 47 (%) | Non-HTAD  n = 175 (%) | p = |
| --- | --- | --- | --- | --- |
| Male | 135 (60.8) | 27 (57.4) | 108 (62.1) | 0.56 |
| Age (years), median (IQR) | 63.8 (53.9–72.2) | 46.3 (36.3–54.5) | 67.0 (59.8–73.5) | <0.001 |
| Age >70 years | 64 (28.8) | 0 (0.0) | 64 (36.8) | <0.001 |
| Arterial hypertension | 207 (93.2) | 38 (80.6) | 169 (96.6) | 0.002 |
| Diabetes | 13 (5.9) | 1 (2.1) | 12 (6.9) | 0.31 |
| Smoking | 80 (36.0) | 6 (12.8) | 74 (42.8) | <0.001 |
| COPD | 44 (19.8) | 3 (6.4) | 41 (23.6) | 0.006 |
| Peripheral vascular disease | 90 (40.5) | 13 (27.7) | 77 (44.0) | 0.047 |
| Severe aortic atherosclerosis | 66 (29.7) | 0 (0.0) | 66 (37.7) | <0.001 |
| Impaired renal function^†^ | 25 (11.3) | 1 (2.1) | 24 (13.7) | 0.025 |
| Preoperative impaired neurological status | 22 (9.9) | 5 (10.6) | 17 (9.7) | 0.04 |
| Preoperative spinal cord ischemia | 8 (3.6) | 0 (0.0) | 8 (4.6) | 0.011 |
| Prior open cardiac or aortic surgery | 46 (20.7) | 22 (46.8) | 24 (13.7) | <0.001 |
| Pathologies  Thoracic aortic aneurysm  Noncomplicated Acute Type A dissection  Complicated Acute Type A dissection  Acute Type B dissection  Chronic dissection | 75 (33.8)  27 (12.2)  40 (18.0)  10 (4.5)  70 (31.5) | 5 (10.6)  3 (6.4)  6 (12.8)  4 (8.5)  29 (61.7) | 70 (40.0)  24 (13.7)  34 (19.4)  6 (3.4)  41 (23.4) | <0.001 |

HTAD, hereditable thoracic aortic disease; IQR, interquartile range; COPD, chronic obstructive pulmonary disease; ^†^Creatinine clearance <50 ml/min;

Supplementary Table S3: Procedural characteristics according to presence/absence of HTAD

| Variable | Entire cohort  n = 222 (%) | HTAD  n = 47 (%) | Non-HTAD  n = 175 (%) | p = |
| --- | --- | --- | --- | --- |
| E-vita open hybrid | 15 (6.8) | 4 (8.5) | 11 (6.3) | 0.53 |
| Thoraflex hybrid | 207 (93.2) | 43 (91.5) | 164 (93.7) |  |
| Simplified FET | 148 (66.7) | 33 (70.2) | 115 (65.7) | 0.55 |
| Ascending procedures  Supracoronary aortic replacement  Bentall procedure  Valve-sparing root replacement | 178 (80.2)  25 (11.3)  18 (8.2) | 32 (68.1)  4 (8.5)  11 (23.4) | 146 (83.4)  21 (12.0)  7 (4.0) | <0.001 |
| Aortic valve replacement | 36 (16.3) | 5 (10.6) | 31 (17.7) | 0.20 |
| Coronary artery bypass grafting | 23 (10.4) | 2 (4.3) | 21 (12.0) | 0.18 |
| Tricuspid valve repair | 8 (3.6) | 0 (0.0) | 8 (4.6) | 0.21 |
| Mitral valve repair | 3 (1.4) | 1 (2.1) | 2 (1.1) | 0.51 |
| Cardiopulmonary bypass time (min), median (IQR) | 243 (203–303) | 265 (220–213) | 236 (198–293) | 0.032 |
| Cross-clamp time (min), median (IQR) | 112 (93–152) | 119 (97–178) | 109 (91–147) | 0.131 |
| Circulatory arrest time (min), median (IQR) | 44 (36–60) | 46 (38–53) | 44 (36–63) | 0.818 |
| Cerebral perfusion time (min), median (IQR) | 67 (57–80) | 68 (60–79) | 67 (55–81) | 0.814 |
| Lowest body temperature (°C), median (IQR) | 25 (24–25) | 25 (24–25) | 25 (24–25) | 0.171 |

HTAD, hereditable thoracic aortic disease; FET, frozen elephant trunk; min, minutes; IQR, interquartile range

Supplementary Table S4: Postoperative complications according to presence/absence of HTAD

| Variable | Entire cohort  n = 222 (%) | HTAD  n = 47 (%) | Non-HTAD  n = 175 (%) | p = |
| --- | --- | --- | --- | --- |
| Transient neurological deficit | 27 (12.2) | 4 (8.5) | 23 (13.1) | 0.36 |
| Permanent neurological deficit | 20 (9.0) | 3 (6.4) | 17 (9.7) | 0.58 |
| Recurrent nerve palsy | 32 (14.4) | 6 (12.8) | 26 (14.9) | 0.10 |
| Spinal cord ischemia | 3 (1.4) | 0 (0.0) | 3 (1.7) | 0.55 |
| Postoperative renal failure | 36 (16.2) | 4 (8.5) | 32 (18.3) | 0.23 |
| Resternotomy for bleeding | 24 (10.8) | 3 (6.4) | 21 (12.0) | 0.48 |
| 30-day mortality | 25 (11.3) | 1 (2.1) | 24 (13.7) | 0.025 |

HTAD, hereditable thoracic aortic disease

Supplementary Table S5: Patient characteristics according to simplified/conventional FET

| Variable | Entire cohort  n = 222 (%) | Simplified FET  n = 148 (%) | Conventional FET  n = 74 (%) | p = |
| --- | --- | --- | --- | --- |
| Male | 135 (60.8) | 88 (59.5) | 47 (63.5) | 0.56 |
| Age (years), median (IQR) | 63.8 (53.9–72.2) | 62.5 (53.4–71.2) | 66.5 (56.0–73.6) | 0.076 |
| Age >70 years | 64 (28.8) | 36 (24.3) | 28 (37.8) | 0.036 |
| Arterial hypertension | 207 (93.2) | 140 (94.6) | 67 (90.5) | 0.24 |
| HTAD | 47 (21.2) | 33 (22.3) | 14 (18.9) | 0.55 |
| Diabetes | 13 (5.9) | 10 (6.8) | 3 (4.1) | 0.55 |
| Smoking | 80 (36.0) | 53 (35.8) | 27 (36.5) | 0.21 |
| COPD | 44 (19.8) | 34 (23.0) | 10 (13.5) | 0.10 |
| Peripheral vascular disease | 90 (40.5) | 65 (34.9) | 25 (33.8) | 0.15 |
| Severe aortic atherosclerosis | 66 (29.7) | 37 (25.0) | 29 (39.2) | 0.032 |
| Impaired renal function^†^ | 25 (11.3) | 15 (10.1) | 10 (13.5) | 0.45 |
| Preoperative impaired neurological status | 22 (9.9) | 11 (7.4) | 11 (14.9) | 0.18 |
| Preoperative spinal cord ischemia | 8 (3.6) | 3 (2.0) | 5 (6.8) | 0.17 |
| Prior open cardiac or aortic surgery | 46 (20.7) | 34 (23.0) | 12 (16.2) | 0.24 |
| Pathologies  Thoracic aortic aneurysm  Noncomplicated Acute Type A dissection  Complicated Acute Type A dissection  Acute Type B dissection  Chronic dissection | 75 (33.8)  27 (12.2)  40 (18.0)  10 (4.5)  70 (31.5) | 56 (37.8)  10 (6.8)  21 (14.2)  9 (6.1)  52 (35.1) | 19 (25.7)  17 (23.0)  19 (25.7)  1 (1.4)  18 (24.3) | <0.001 |

FET, frozen elephant trunk; IQR, interquartile range; HTAD, hereditable thoracic aortic disease; COPD, chronic obstructive pulmonary disease; ^†^Creatinine clearance <50 ml/min;

Supplementary Table S6: Procedural characteristics according to simplified/conventional FET

| Variable | Entire cohort  n = 222 (%) | Simplified FET  n = 148 (%) | Conventional FET  n = 74 (%) | p = |
| --- | --- | --- | --- | --- |
| E-vita open hybrid | 15 (6.8) | 4 (2.7) | 11 (14.9) | <0.001 |
| Thoraflex hybrid | 207 (93.2) | 144 (97.3) | 63 (85.1) |  |
| Ascending procedures  Supracoronary aortic replacement  Bentall procedure  Valve-sparing root replacement | 178 (80.2)  25 (11.3)  18 (8.2) | 124 (83.8)  12 (8.1)  11 (7.4) | 54 (73.0)  13 (17.6)  7 (9.5) | 0.19 |
| Aortic valve replacement | 36 (16.3) | 20 (13.5) | 16 (21.6) | 0.34 |
| Coronary artery bypass grafting | 23 (10.4) | 15 (10.1) | 8 (10.8) | 0.90 |
| Tricuspid valve repair | 8 (3.6) | 7 (4.7) | 1 (1.4) | 0.27 |
| Mitral valve repair | 3 (1.4) | 3 (2.0) | 0 (0.0) | 0.55 |
| Cardiopulmonary bypass time (min), median (IQR) | 243 (203–303) | 230 (195–279) | 271 (227–334) | <0.001 |
| Cross-clamp time (min), median (IQR) | 112 (93–152) | 101 (88–128) | 148 (109–197) | <0.001 |
| Circulatory arrest time (min), median (IQR) | 44 (36–60) | 39 (35–74) | 68 (51–92) | <0.001 |
| Cerebral perfusion time (min), median (IQR) | 67 (57–80) | 62 (53–72) | 84 (70–113) | <0.001 |
| Lowest body temperature (°C), median (IQR) | 25 (24–25) | 25 (24–25) | 24 (23–25) | 0.008 |

HTAD, hereditable thoracic aortic disease; FET, frozen elephant trunk; min, minutes; IQR, interquartile range

Supplementary Table S7: Postoperative complications according to simplified/conventional FET

| Variable | Entire cohort  n = 222 (%) | Simplified FET  n = 148 (%) | Conventional FET  n = 74 (%) | p = |
| --- | --- | --- | --- | --- |
| Transient neurological deficit | 27 (12.2) | 18 (12.2) | 9 (12.2) | 0.65 |
| Permanent neurological deficit | 20 (9.0) | 10 (6.8) | 10 (13.5) | 0.097 |
| Recurrent nerve palsy | 32 (14.4) | 14 (9.5) | 18 (24.3) | <0.001 |
| Spinal cord ischemia | 3 (1.4) | 2 (1.3) | 1 (1.4) | 0.46 |
| Postoperative renal failure | 36 (16.2) | 18 (12.2) | 18 (24.3) | 0.023 |
| Resternotomy for bleeding | 24 (10.8) | 10 (6.8) | 14 (18.9) | 0.008 |
| 30-day mortality | 25 (11.3) | 11 (7.4) | 14 (18.9) | 0.011 |

FET, frozen elephant trunk; min, minutes;

| Pathology | Total number of secondary interventions, n = | Pre-FET planned,  n = (% of total interv.) | Due to disease progression,  n = (% of total interv.)  elective urgent | |
| --- | --- | --- | --- | --- |
| Aneurysm | 29 | 19 (65.5) | 8 (27.6) | 2 (6.9) |
| Acute aortic dissection | 24 | 1* (4.2) | 22 (91.7) | 1 (4.2) |
| Chronic aortic dissection | 30 | 8 (26.7) | 16 (53.3) | 6 (20.0) |

Supplementary Table S8: Secondary distal aortic interventions as preoperatively planned second step procedure or due to disease progression

FET, frozen elephant trunk; *Acute Type B dissection with large false lumen aneurysm in the descending aorta and entry distally of the expected FET landing zone
